# Supplementary material for: Insights from single-strain and mixed culture experiments on the effects of heatwaves on freshwater flagellates
Source: PeerJ. 2024 Sep 12;12:e17912. doi: 10.7717/peerj.17912 (PMC11402338; doi:10.7717/peerj.17912)
Supplement: Supplemental Information 1 [file peerj-12-17912-s001.docx]

| **compounant** | **Quantity in IB medium**  [mg.l^-1^ ] | **Quantity in NSY medium**  mg.l^-1^ |
| --- | --- | --- |
| MgSO_4_ · 7H_2_O | 75 | 75 |
| Ca(NO_3_)_2_ · 4H_2_O | 43 | 43 |
| NaHCO_3_ | 16 | 16 |
| KCL | 5 | 5 |
| K_2_HPO_4_ | 2,8 | 2,8 |
| Na_2_EDTA · 2H_2_O | 4,4 | 4,4 |
| FeCl_3_ · 6H_2_O | 3,2 | 3,2 |
| H_3_BO_3_ | 1 | 1 |
| MnCl_2_ · 4H_2_O | 0,2 | 0,2 |
| ZnSO_4_ · 7H_2_O | 0,02 | 0,02 |
| CuSO_4_ · 6H_2_O | 0,01 | 0,01 |
| CoCl_2_ · 6H_2_O | 0,01 | 0,01 |
| Na_2_MoO_4_ · 2H_2_O | 0,006 | 0,006 |
| Yeast Extract | / | 1000 |
| Soyotone | / | 1000 |
| Nutrient Broth | / | 1000 |
