# Supplementary figures and images for: Insights from single-strain and mixed culture experiments on the effects of heatwaves on freshwater flagellates
Source: PeerJ. 2024 Sep 12;12:e17912. doi: 10.7717/peerj.17912 (PMC11402338; doi:10.7717/peerj.17912)

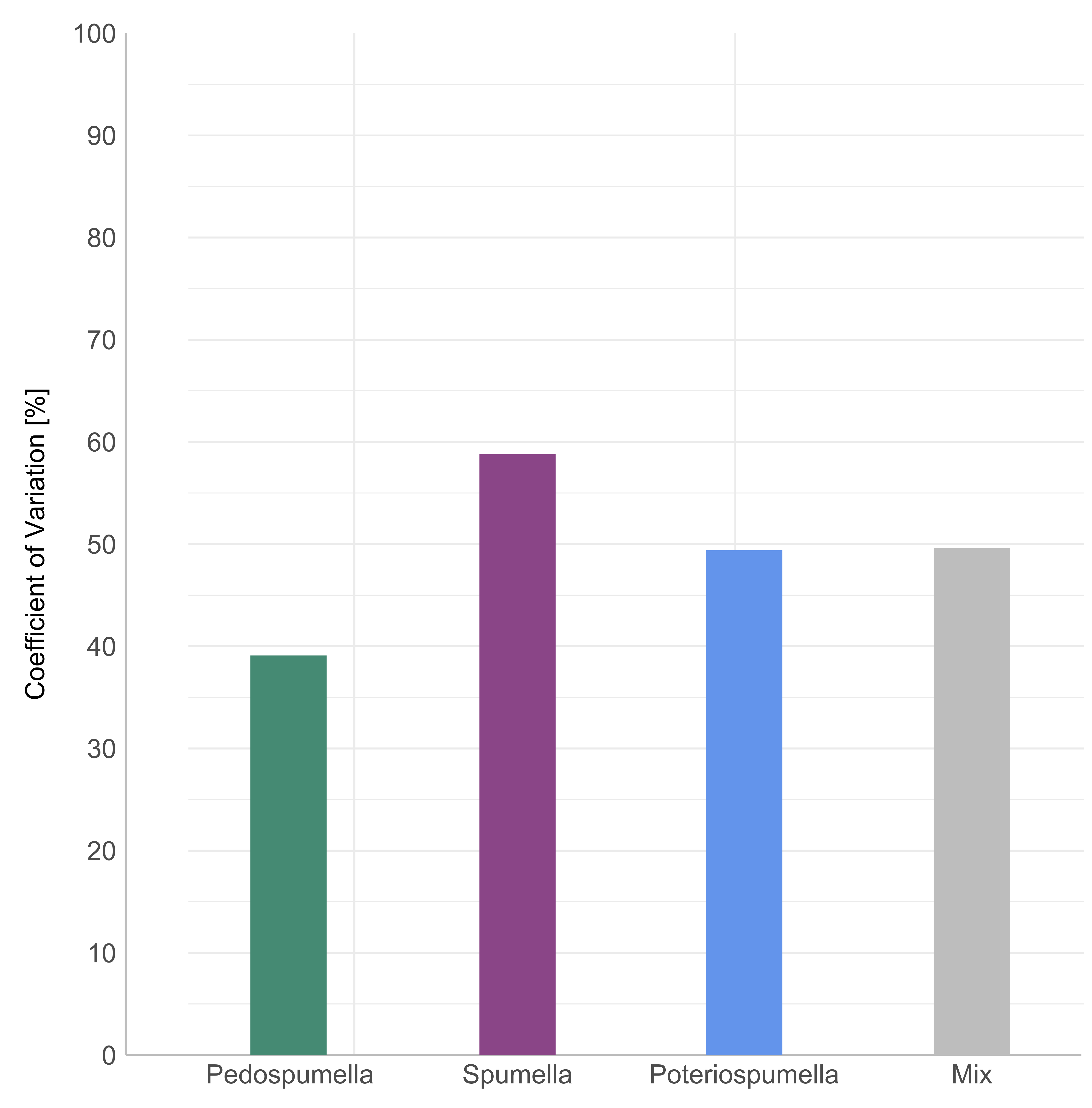

Supplement: Supplemental Information 2 — Coefficient of variation calculated from the bacterial abundances in single-strain cultures of Pedospumella encystans, Spumella rivalis, Poteriospumella lacustris and mixed cultures containing all three strains. [file peerj-12-17912-s002.png]
